# Association of preoperative EpCAM Circulating Tumor Cells and peripheral Treg cell levels with early recurrence of hepatocellular carcinoma following radical hepatic resection

**DOI:** 10.1186/s12885-016-2526-4

**Published:** 2016-07-20

**Authors:** Yan Zhou, Beili Wang, Jiong Wu, Chunyan Zhang, Yiwen Zhou, XinRong Yang, Jian Zhou, Wei Guo, Jia Fan

**Affiliations:** Liver Cancer Institute, Fudan University, 136 Yi Xue Yuan Road, Shanghai, 200032 People’s Republic of China; Department of Laboratory Medicine, Zhongshan Hospital, Fudan University, 136 Yi Xue Yuan Road, Shanghai, 200032 People’s Republic of China

**Keywords:** Hepatocellular carcinoma, Tumor recurrence, Circulating tumor cells, Epithelial cell adhesion molecule, Regulatory T cells

## Abstract

**Background:**

This study was carried out to determine the prognostic significance of preoperative peripheral epithelial cell adhesion molecule- positive (*EpCAM*^+^) circulating tumor cell (CTC) and T regulatory (Treg) cell levels in hepatocellular carcinoma (HCC) patients for the prediction of postoperative recurrence following curative resection.

**Methods:**

A total of 49 patients about to undergo curative resection for HCC were recruited into the study. PCR and FACS were used to detect the preoperative levels of *EpCAM*^mRNA+^ CTCs and CD4^+^CD25^+^Foxp3^+^ Treg cells. The prognostic value of *EpCAM*^mRNA+^ CTCs, CD4^+^CD25^+^Foxp3^+^ Treg cells, and other clinicopathological factors were analyzed by applying the Kaplan–Meier method and the multivariate Cox proportional hazards model.

**Results:**

The number of *EpCAM*^mRNA+^ CTCs and Treg/CD4^+^ cells showed significant correlation as prognostic factors of postoperative HCC recurrence: *EpCAM*^mRNA+^ CTC ≥ 2.22 (P = 0.001) and Treg/CD4^+^ ≥ 5.07 (*P* = 0.045), with *EpCAM*^mRNA+^ CTC ≥ 2.22 (*P* = 0.003, HR = 6.668) being the most important indicator. Patients with high CTC/Treg levels showed a significantly higher risk of developing postoperative HCC recurrence than those with low CTC/Treg levels (66.7 % vs. 10.3 %, *P* < 0.001). The high CTC/low Treg group also presented higher 1-year recurrence rates compared with the low CTC/low Treg level group (50.0 % vs. 10.3 %, *P* = 0.004).

**Conclusions:**

Elevated *EpCAM*^mRNA+^ CTC and Treg/CD4^+^ levels were associated with early recurrence of HCC, indicative of poor clinical outcome. The combined detection of *EpCAM*^mRNA+^ CTC and Treg/CD4^+^ may therefore provide a novel prognostic predictor for HCC patients.

**Electronic supplementary material:**

The online version of this article (doi:10.1186/s12885-016-2526-4) contains supplementary material, which is available to authorized users.

## Background

Hepatocellular carcinoma (HCC) is the fifth most common malignancy worldwide, and is ranked second in global cancer-related mortality. The high incidence and poor prognosis of HCC are current focuses of clinical research [1]. The main treatments for HCC include surgical resection and liver transplantation. However, the tumor recurrence rate exceeds 70 % after 5 years following resection, and recurrence is considered the main contributor to mortality [2]. Imaging tests and pathological examination are limited in terms of accuracy and sensitivity, while common serum markers display poor diagnostic performance [3]. It is therefore critical to find robust prognostic biomarkers with which to monitor postoperative recurrence for HCC patients [4].

The progression of a tumor consists of two stages: growth of the primary tumor and development of distant metastases. Circulating tumor cells (CTCs) spread from the primary tumor sites or the metastases into the peripheral blood supply, and possess characteristics of stem cells combined with invasive ability. Distant metastases induced by CTC invasion are believed to be responsible for the majority cases of recurrence and cancer-related deaths. Therefore, isolation and detection of CTCs will help us to understand the processes of early metastasis and recurrence, and the aggressiveness of tumors [5].

Epithelial cell adhesion molecule-positive (EpCAM^+^) CTCs are a proven independent risk factor for HCC recurrence in our previous study [6], while immune suppressive CD4^+^CD25^+^ regulatory T cells (Treg) intra-nuclear expressing Foxp3^+^ are associated with tumor immune tolerance and immune escape [7]. Treg cell proliferation is known to be significantly associated with tumor invasion and poor prognosis [7], and increased proportions of Foxp3^+^ Treg cells were shown to be an important predictor for the high recurrence and poor survival rates of HCC patients [8].

The prognostic significance of CTC or Treg cells alone for HCC recurrence has therefore already been investigated; however, the prognostic value of CTCs in combination with Tregs has not yet been established. The objective of our study was therefore to determine the prognostic significance of preoperative EpCAM^+^ CTCs and Treg cells population levels for recurrence in HCC patients following curative resection, and to explore the interaction between EpCAM^+^ CTCs and the tumor immune microenvironment.

## Methods

### Patients

From March to June 2012, 49 HCC patients undergoing curative resection at the Zhongshan Hospital were recruited (36 males and 13 females), with a median age of 50 years (range: 37 to 83). According to Child-Pugh score criteria, 48 patients were classified as grade A, and one as grade B. All cases enrolled had to fulfill the following criteria: (i) Hepatitis B virus-related HCC with pathological diagnosis; (ii) about to receive curative resection; (iii) no history of blood transfusion, acquired immunologically mediated disease or any anti-tumor treatment within the preceding 6 months. This study adopted the Barcelona Clinic Liver Cancer (BCLC) staging system and the Edmondson-Steiner grading system. Fifty healthy volunteers were recruited as the control group (35 males and 15 females). All patients provided informed consent before sample collection.

Approval for the use of human subjects was obtained from the Research Ethics Committee of Zhongshan Hospital, and informed consent was obtained from each individual enrolled in this study.

### Specimen collection

A peripheral blood sample (6 mL each) was collected into an EDTA-K2 anticoagulant tube (BD Biosciences, USA in the morning on the day of surgery before the operation. Prior to this, the first 6 mL of blood was discarded to avoid epithelial cell contamination. RNA extraction and reverse transcription were completed within 8 h following collection (details below). Samples of cDNA were preserved at −20 °C. All the patients received curative resection and the common operation time was about 2–3 h and the average bleeding was 300 ml.

### Apparatus and reagents

The monocyte isolation kit used was Ficoll-Paque Plus (GE Healthcare, USA). CD45 cells were isolated with RosetteSep Human CD45 Depletion Cocktail (Stemcell Technologies, Canada). Other equipment used included the RNA extraction kit, RNeasy Mini Kit (Qiagen, Germany), the QuantiTect Reverse Transcription kit, the Human Regulatory T cell Staining Kit (eBioscience, USA), the LightCycler 480 Real-time PCR system (Roche, Switzerland) and the FACS Calibur flow cytometry system (BD Biosciences, USA).

### *EpCAM*^mRNA+^ CTC detection and qRT-PCR

CTC detection was processed by a negative enrichment and quantitative real time polymerase chain reaction (qRT-PCR) based platform [9]. A peripheral blood sample was collected for each patient (5 ml). Target cells were first negative enriched by RosetteSep Human CD45 Depletion Cocktail (StemCell, Canada), which to remove leukocyte impureness [9]. After enrichment, messenger RNA (mRNA) was extracted from the target cells with an RNeasy Mini Kit and then reverse transcribed into cDNA using the QuantiTect Reverse Transcri EpCAM ption kit. All protocols were according to manufacturer’s instructions. qRT-PCR analysis of and β-actin (as an internal control) transcripts were performed using the Light Cycler 480 platform (Roche Diagnostics, Germany) with fluorescent Taqman methodology. PCR reactions were performed using the following conditions: 2 min at 50 °C and 2 min at 95 °C, followed by 45 cycles at 95 °C for 30 s and 60 °C for 30 s. Florescent detection was performed at 60 °C, and three replicates were carried out for each sample. Invitrogen (nvitrogen, USA) synthesized the primers and probe segments. The forward primer :5′-CTCGCGTTCGGGCTTCT-3′, the reverse primer: 5′- TGTAGTTTTCACAGACACATTCTTCCT-3′, and the probe [6FAM] ACGGCGACTTTTGCCGCAGCTTA-MRA were used for analysis of EpCAM expression. The forward primer: 5′-TGGCATTGCCGACAGGAT-3′, the reverse primer: 5′-CTCAGGAGGAGCAATGATCTTGAT-3′, and the probe [6FAM] -ATCACTGCCCTGGCACCCAGCATA-MRA were used for analysis of β-actin expression. All primers and probes were designed and synthesized by the Life Technology Corporation (Invitrogen, USA).

Gene expression levels were calculated with the following equations:

- 2^−ΔΔCT^ [ΔCT = Ct (EpCAM) – Ct (β-actin), and ΔΔCT = ΔCT − Ct(calibrator),
- where Ct (calibrator) stands for the mean ΔCT of the 50 healthy volunteers [9, 10].

### Detection of lymphocyte subgroups

Two sets of four-color florescent antibody, CD3/CD8/CD45/CD4 (BD Biosciences) and CD3/CD16^+^ CD56/CD45/CD19 (BD Biosciences), were added (20 μl each) into two separate flow cytometry tubes. The sample (50 μl whole blood each) was added to each tube, followed by incubation at room temperature (RT) under darkness for 15 min. After adding 0.45 ml erythrocytolysin (BD Biosciences), the solution was incubated for another 10 min. The solution was then centrifuged for 5 min at 1200 rpm. The supernatant was discarded and the pellet was washed twice with 2 ml PBS. After resuspension in 0.4 ml phosphate-buffered saline (PBS), the sample was loaded for flow cytometry analysis. Data analyses were performed with MultiSET software (BD Biosciences). Measurements included percentages of B cells (CD19^+^), T cells (CD3^+^), CD4^+^ T cells, CD8^+^ T cells and NK cells (CD16^+^CD56^+^) and the ratio of CD4^+^/CD8^+^ T cells.

### Detection of CD4^+^CD25^+^Foxp3^+^ Tregs

After the addition of 20 μl anti-CD4-FITC/anti-CD25-APC (eBioscience) and the relevant isotype control antibody (IgG1Ƙ-FITC and IgG1Ƙ-APC, respectively; eBioscience) into two separate flow cytometry tubes, 100 μL whole blood sample was added, followed by incubation at 4 °C for 30 min in darkness. After the erythrocytolysis step (as above), each tube was supplemented with 1 ml permeabilization reagent and incubated at 4 °C for 60 min. After washing with PBS, 100 μl mouse serum was added to the solution and the mixture was incubated at RT under darkness for 15 min. Intracellular antibody 20 μl anti-Foxp3-PE, and isotype control IgG2a-K-PE (both from eBioscience), were each added, and the resulting solution was incubated at RT under darkness for another 30 min. After resuspension in PBS, the sample was loaded for flow cytometry analysis. Measurements included proportions of CD4^+^CD25^+^Foxp3^+^ T cells (Tregs) in total lymphocytes, CD8^+^ T cells, CD4^+^ T cells and CD3^+^ T cells.

### Follow-up for HCC recurrence

All patients had postoperative follow-ups [11]. Time to recurrence (TTR) was defined as the period from curative excision to diagnosis of HCC recurrence (including intrahepatic recurrence and extrahepatic metastasis) based on MRI and serum AFP levels [12, 13]. Early recurrence was defined as recurrence within 12 months following excision [14].

### Statistical analysis

Prognostic cut-off values were determined using X-tile 3.6.1 software [15]. All statistical analyses were performed using SPSS 17.0. Categorical data and measurement data were assessed with the *χ*^2^ test and the *t*-test, respectively. Prognostic factors for early recurrence were evaluated with univariate analysis and multivariate COX regression analysis. The associations between TTR and the prognostic factors were assessed with Kaplan-Meier survival analysis, and the inter-curve differences were assessed with the log-rank test. P values <0.05 were considered statistically significant.

## Results

### Patient characteristics

The characteristics of the study participants were listed in Additional file 1: Table S1. The cohorts were well matched for serum AFP, age, and sex overall. There were 44 (89.8 %) patients with early HCC (BCLC 0-A) and 14 (28.6 %) patients with early recurrence. Majority of patients had Child-Pugh score A with multiple tumor.

### *EpCAM*^mRNA+^ CTCs and Treg/CD4^+^ cells definition

According to previous studies, the prognostic cut-off value for HCC recurrence of *EpCAM*^mRNA+^ CTCs (2^−ΔΔCT^) was 2.0 [9]. In our study, the optimal prognostic cut-off values calculated by X-tile 3.6.1 software for *EpCAM*^mRNA+^ CTCs (2^−ΔΔCT^) and Treg/CD4^+^ (%) were 2.22 and 5.07, respectively. The original data of flow cytometry analysis for Tregs was shown in Additional file 2: Figure S1.

### Association of clinical characteristics and pathological factors with early recurrence

**Fig. 1:**
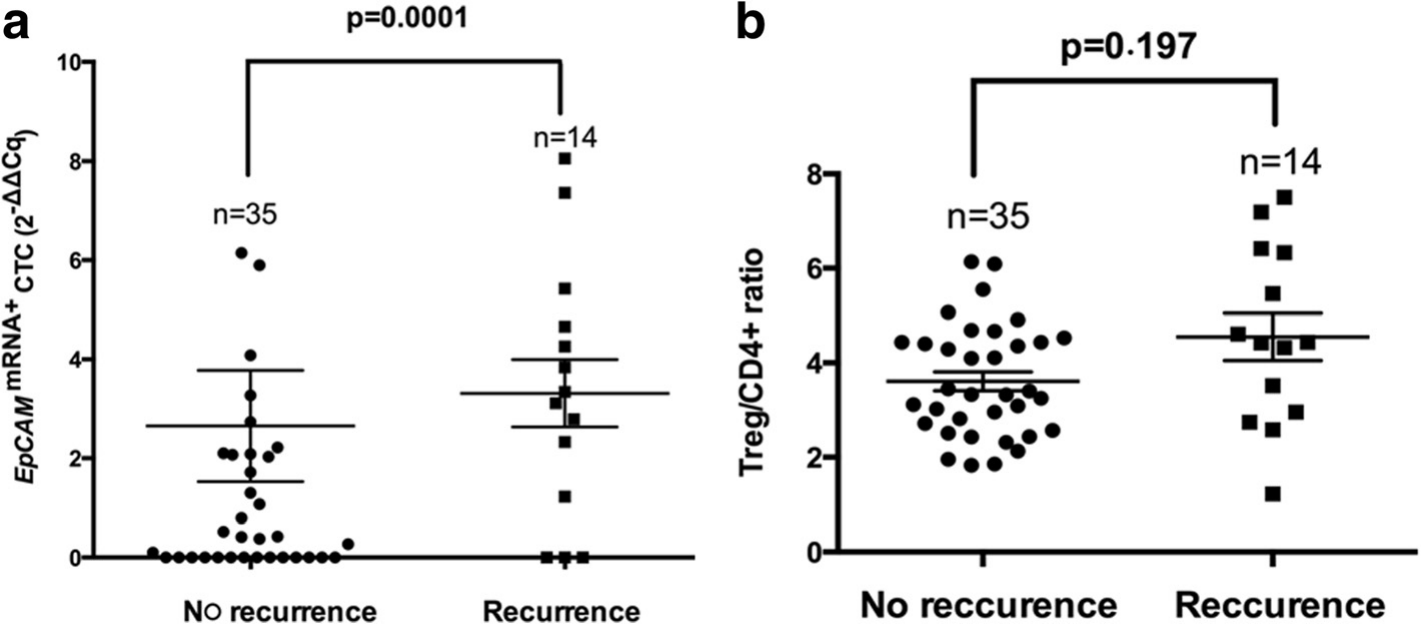
Expression of *EpCAM* ^mRNA+^ CTC and Treg/CD4+ in HCC recurrence vs no recurrence group. **a**, Based on 2^−ΔΔ*C*^_q_ algorithm transformation, the relative *EpCAM* mRNA expression of CTC in HCC patients with or without tumor recurrence after resection. *EpCAM* ^mRNA+^ CTC showed significantly higher in recurrence patients (*p* = 0.001); **b**, The high ratio of Treg/CD4^+^ was observed d in recurrence group (*p* = 0.0197)

*EpCAM*^mRNA+^ CTC (*P* < 0.001; Fig. 1a) and Treg/CD4^+^ ratio (*P* = 0.02; Fig. 1a, Additional file 3: Table S2) was observed to be significantly higher in the postoperative recurrence in recurrence group (Table 1). In addition, Satellite lesion showed inverse correlation with early recurrence. There was a positive association between vascular invasion and early recurrence in HCC patients (Table 1).

**Table 1:** Association of clinical characteristics and pathological factors with early recurrence

Variables		Early recurrence		*P*
		No	Yes	
Age	≤50	15	8	0.365
	>50	20	6	
Sex	male	26	10	0.838
	female	9	4	
HBsAg	Negative	7	2	0.641
	Postive	28	12	
HBeAg	Negative	27	9	0.357
	Postive	8	5	
HBVDNA	<5*10^2^	21	10	0.453
	>5*10^2^	14	4	
ALT (U/L)	≤40	30	9	0.412
	>40	5	5	
AFP (ng/ml)	≤400	23	10	0.768
	>400	12	4	
EpCAM ^mRNA+^ CTC (2-ΔΔCq)	Low (<2.22)	28	4	<0.001*
	High (≥2.22)	7	10	
Treg/CD4+ (%)	<5.07	31	9	0.020*
	≥5.07	4	5	
Child-Pugh stage	A	34	14	0.523
	B	1	0	
Liver cirrhosis	No	9	3	0.753
	Yes	26	11	
Tumor number	Single	33	11	0.101
	Multiple	2	3	
Tumor size (cm)	≤5	21	8	0.854
	>5	14	6	
Tumor encapsulation	Complete	16	5	0.523
	None	19	9	
Satellite lesion	No	34	10	0.007*
	Yes	1	4	
Vascular invasion	No	21	4	0.047*
	Yes	14	10	
Edmondson stage	I-II	22	9	0.925
	II-IV	13	5	
BCLC stage	0 + A	33	11	0.101
	B + C	2	3	

*Abbreviations*: *ALT* alanine transaminase, *HBsAg* Hepatitis B surface antigen, *AFP* alphafetoprotein, *HBeAg* Hepatitis B e antigen. *p* value of < 0.05 was considered statistically-significant

Association of *EpCAM*^mRNA+^ CTCs and Treg/CD4^+^ cells with clinical characteristics.

**Fig. 2:**
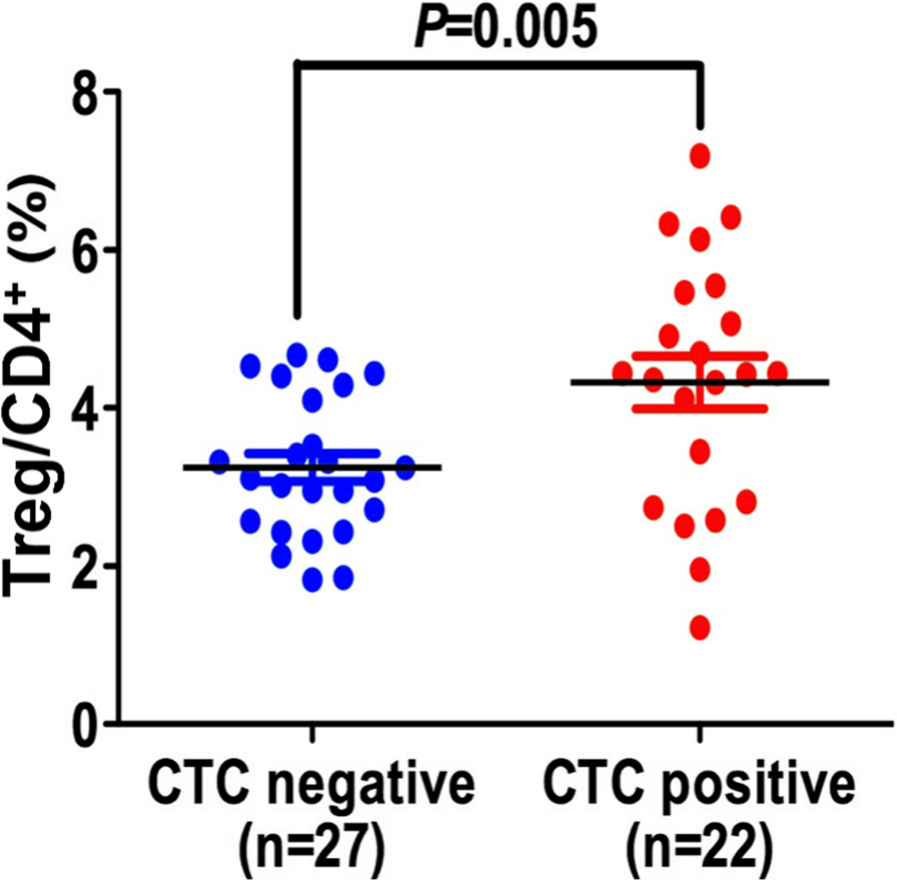
Association between EpCAM^+^ CTC and Treg/CD4^+^ ratio. Distribution of Treg/CD4^+^ ratio in *EpCAM* ^*mRNA+*^ -positive and negative group. The cutoff to discriminate between CTC-positive and -negative was set at 2.0 [9]

Clinical characteristics and associations with *EpCAM*^mRNA+^ CTCs and Treg/CD4^+^ were analyzed using the χ ^2^ test and the *t*-test for two subgroups of HCC patients according to the calculated cut-off values (Tables 2 and 3). Treg/CD4^+^ ratio was elevated in the *EpCAM*^mRNA+^ CTC ≥ 2.22 group (*P* = 0.026; Table 4, Fig. 2). A rising rate of vascular invasion within the *EpCAM*^mRNA+^ CTC ≥ 2.22 group was also confirmed (*P* = 0.027, Table 2). Patients within the Treg/CD4^+^ ≥ 5.07 % group were more likely to be younger (age ≤ 50 years old; *P* = 0.040) and male (*P* = 0.046, Table 3), and displayed an increased number of multiple satellite focuses in pathological specimens (*P* = 0.011, Table 3).

**Table 2:** Association of EpCAM^+^ CTCs with clinical characteristics

Clinical characteristics		CTC (2^-ΔΔCq^)		*P*
		Low (<2.22)	High (≥2.22)	
Age (y)	≤50	13	10	0.224
	>50	19	7	
Sex	Female	9	4	0.729
	Male	23	13	
HBsAg	Negative	7	2	0.384
	Postive	25	15	
HBeAg	Negative	24	12	0.586
	Postive	8	5	
HBVDNA	<5*10^2^	21	10	0.703
	>5*10^2^	11	7	
Child-Pugh score	A	32	16	0.166
	B	0	1	
Liver cirrhosis	No	8	4	0.909
	Yes	24	13	
ALT (U/L)	≤40	19	14	0.103
	>40	13	3	
AFP (ng/ml)	≤400	28	11	0.060
	>400	4	6	
Tumor number	Single	30	4	0.210
	Multiple	2	3	
Tumor size (cm)	≤5	18	11	0.566
	>5	14	6	
Tumor encapsulation	Complete	15	6	0.436
	None	17	11	
Satellite lesion	No	30	4	0.210
	Yes	2	3	
Vascular invasion	No	20	5	0.027*
	Yes	12	12	
Edmondson stage	I-II	20	11	0.879
	II-IV	12	6	
BCLC stage	0 + A	30	14	0.210
	B + C	2	3	
Treg/CD4^+^ (%)	<5.07	29	11	0.026*
	≥5.07	3	6	
Early recurrence	No	28	7	0.001*
	Yes	4	10	

*p* value of < 0.05 was considered statistically-significant

**Table 3:** Association of Treg/CD4^+^ with clinical characteristics

Clinical characteristics		Treg/CD4^+^ <5.07 %	Treg/CD4 ≥ 5.07 %	*P*
Age (y)	≤50	16	7	0.040*
	>50	24	2	
Sex	Male	13	0	0.046*
	Female	27	9	
HBsAg	Negative	8	1	0.534
	Postive	32	8	
HBeAg	Negative	30	6	0.609
	Postive	10	3	
HBVDNA	<5*10^2^	25	6	0.815
	>5*10^2^	15	3	
Child-Pugh score	A	39	9	0.632
	B	1	0	
Liver cirrhosis	No	10	2	0.861
	Yes	30	7	
ALT (U/L)	≤75	26	7	0.460
	>75	14	2	
AFP (ng/ml)	≤400	32	7	0.881
	>400	8	2	
Tumor number	Single	36	8	0.921
	Multiple	4	1	
Tumor size (cm)	≤5	25	4	0.319
	>5	15	5	
Tumor encapsulation	Complete	17	4	0.915
	None	23	5	
Satellite lesion	No	38	6	0.011*
	Yes	2	3	
Vascular invasion	No	22	3	0.240
	Yes	18	6	
Edmondson stage	I-II	25	6	0.815
	II-IV	15	3	
BCLC stage	0 + A	37	7	0.187
	B + C	3	2	

*p* value of < 0.05 was considered statistically-significant

**Table 4:** Association between EpCAM^+^ CTC and Treg/CD4^+^

		EpCAM mRNA + CTC(2-ΔΔCT)		***P***
		Low (<2.22)	High (≥2.22)	
Treg/CD4^+^ (%)	<5.07	29	11	0.026*
	>5.07	3	6	

*p* value of < 0.05 was considered statistically-significant

### Prognostic model for HCC recurrence

Prognostic models for HCC recurrence were determined with univariate analysis and multivariate Cox analysis. Univariate analysis revealed significant prognostic factors for early recurrence in the 49 HCC patient cohort, including *EpCAM*^mRNA+^ CTC ≥ 2.22 (*P* = 0.001), Treg/CD4^+^ ≥ 5.07 (*P* = 0.045), and satellite lesions (*P* = 0.004). Moreover, multivariate Cox analysis revealed that *EpCAM*^mRNA+^ CTC ≥ 2.22 (*P* = 0.003, HR = 6.668, Table 5) and satellite lesions (*P* = 0.019, HR = 5.917, Table 5) were dependent prognostic factors. Of these three factors, *EpCAM*^mRNA+^ CTC ≥ 2.22 was determined to be the key prognostic factor (Table 5).

**Table 5:** Analyses of risk factors for HCC recurrence

	Univariate analysis		Multivariate analysis	
	HR (95 % CI)	*P*	HR (95 % CI)	*P*
Age (>50y vs. ≤ 50y)	0.626 (0.217–1.804)	0.385	N.A.	
Gender (Male vs. Female)	0.886 (0.278–2.827)	0.838	N.A.	
HBsAg (Positive vs. Negative)	1.433 (0.321–6.406)	0.638	N.A.	
Cirrhosis (Positive vs. Negative)	1.323 (0.369–4.743)	0.668	N.A.	
Child-Pugh grade (B vs. A)	0.048 (0.000–23944)	0.700	N.A.	
ALT (>40 U/L vs. ≤ 40 U/L)	0.783 (0.246–2.497)	0.679	N.A.	
AFP (>400 ng/ml vs. ≤ 400 ng/ml)	2.258 (0.756–6.743)	0.145	N.A.	
Tumor number (multiple vs. one)	3.570 (0.998–12.896)	0.052	N.A.	
Tumor size (>5 cm vs. ≤5 cm)	1.094 (0.379–3.154)	0.868	N.A.	
Tumor encapsulation (Yes vs. No)	1.425 (0.477–4.252)	0.526	N.A.	
Satellite lesion (Yes vs. No)	5.726 (1.771–18.509)	**0.004**	5.917 (1.342–26.078)	**0.019**
Vascular invasion (Yes vs. No)	2.943 (0.922–9.399)	0.068	N.A.	
Edmondson classification (III-IV vs. I-II)	1.057 (0.354–3.155)	0.921	N.A.	
BCLC stage (B + C vs. 0 + A)	3.496 (0.966–12.646)	0.056	N.A.	
EpCAM^+^ CTC 2^-ΔΔCq^ (≥2.22 vs. < 2.22)	6.580 (2.056–21.055)	**0.001**	6.668 (1.943–22.883)	**0.003**
Treg/CD4^+^(≥5.07 vs. < 5.07)	2.993 (0.998–8.947)	**0.045**	0.825 (0.196–3.468)	0.792

### Survival analysis

**Fig. 3:**
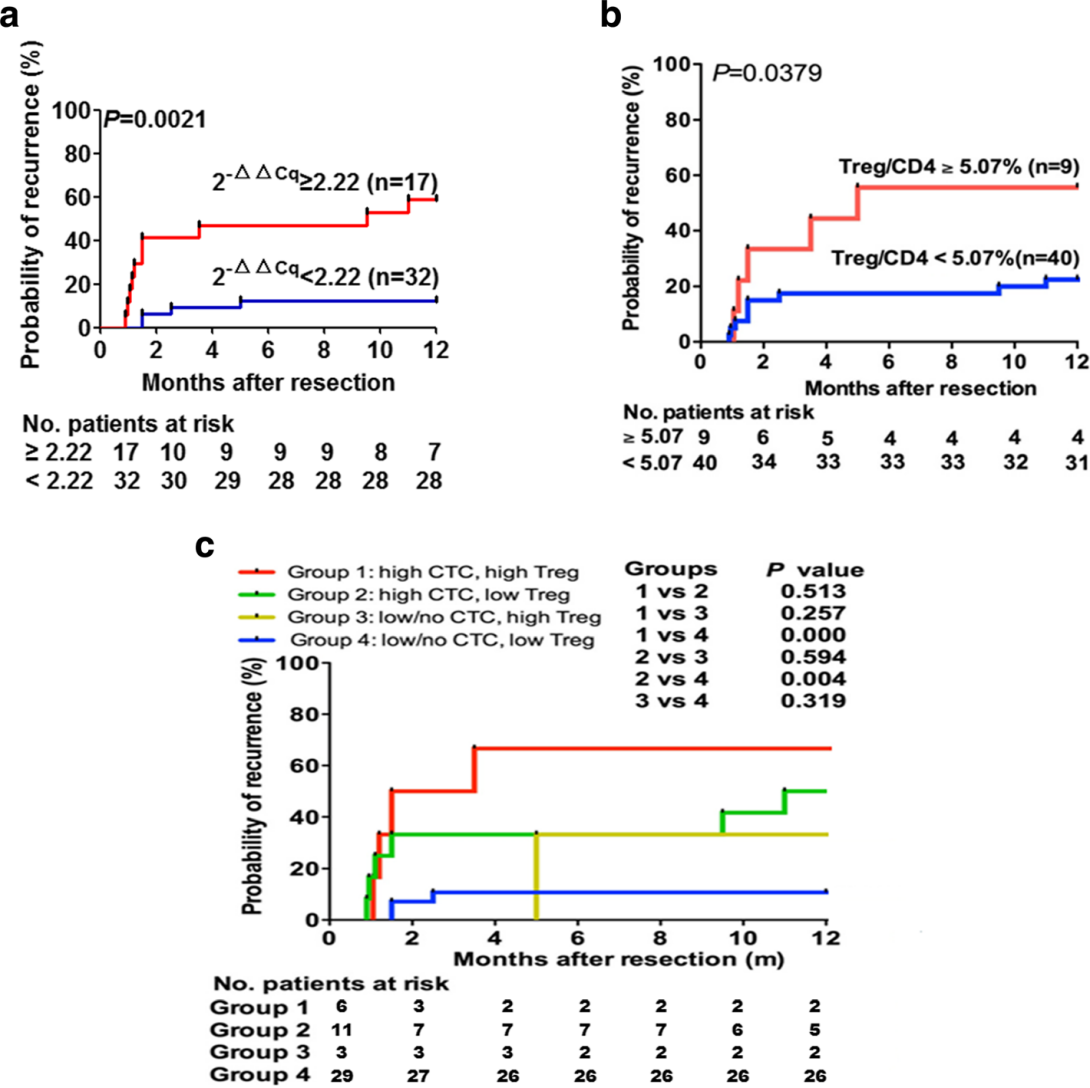
The Prognostic significance of EpCAM ^mRNA+^ CTC and Treg/CD4^+^ ratio in HCC patients. **a**. Kaplan–Meier analysis of HCC patients receiving curative resection treatment according to relative preoperative *EpCAM* expression <2.22 or ≥2.22 (2^−ΔΔC^_q_ algorithm transformation). **b**. Kaplan–Meier analysis of patients with HCC undergoing curative resection according to preoperative Treg/CD4+ <5.07 % or ≥5.07 %. **c**. Prognostic significance of combination of *EpCAM* ^mRNA+^ CTC and Treg/CD4^+^ load with respect to time to recurrence in patients with high *EpCAM* ^mRNA+^ CTC (≥2.22) and high Treg/CD4^+^(≥5.07 %), high *EpCAM* ^mRNA+^ CTC(<2.22) and low Treg/CD4^+^(<5.07 %), low/no *EpCAM* ^mRNA+^ CTC (<2.22) and high Treg/CD4^+^(≥5.07 %), and low/no *EpCAM* ^mRNA+^ CTC (<2.22) and low Treg/CD4^+^(<5.07 %)

The 1-year recurrence rates were 12.5 % and 58.8 % in the *EpCAM*^mRNA+^ CTC < 2.22 group and *EpCAM*^mRNA+^ CTC ≥ 2.22 group, respectively (*P* = 0.002; Fig. 3a); and similarly 22.5 % and 55.6 % in the Treg/CD4^+^ < 5.07 group and Treg/CD4^+^ ≥ 5.07 group, respectively (*P* = 0.002; Fig. 3b). Patients were categorized into four groups according to their combined levels of *EpCAM*^mRNA+^ CTC and Treg/CD4^+^: HH group (high CTC and high Treg levels), HL group (high CTC and low Treg levels), LH group (low CTC and high Treg levels) and LL group (low CTC and low Treg levels). There was a statistically significant difference between the 1-year recurrence rates in the HH group and the LL group (66.7 % vs.10.3 %, *P* < 0.001; Fig. 3c). The recurrence rate in the HL group was 46.4 % higher than in the LL group (50.0 % vs. 10.3 %, *P* = 0.004; Fig. 3c).

## Discussion

Currently, although surgical resection has greatly improved survival rates among HCC patients, HCC remains one of the leading causes of malignancy-related mortality worldwide with a prognosis that is still far from satisfactory, mainly due to increasing post-operative recurrence [16]. At present, predictions of recurrence are mainly based on imaging or biomarkers, which have limits to reflect the dynamic changes in tumor microenvironment. It was reported that predictions of recurrence and metastasis of HCC are influenced by characteristics of both tumor cells and the tumor immune microenvironment [17]. In our previous studies, molecular markers expressed on circulating tumor cells were found to be closely associated with early diagnosis and early recurrence of HCC [18]. Moreover, CD4^+^CD25^+^Foxp3^+^Tregs are considered as suppressors in immune surveillance and anti-tumor immunity, which are also proved associated with HCC invasiveness [19]. However, no previous publications have evaluated prognostic performance of circulating tumor cells and CD4^+^CD25^+^Foxp3^+^Tregs. Here we first detected the combined effect of circulating tumor cells and its immune environment on hepatocellular carcinoma.

Based on the optimal prognostic cut-off values for *EpCAM*^mRNA+^ CTC (2 − ΔΔCT) of 2.22, and Treg/CD4+ (%) of 5.07, as calculated using X-tile software, we found that the recurrence rate was elevated in the *EpCAM*^mRNA+^ CTC (2 − ΔΔCT) ≥ 2.22 group (*P* = 0.001) and the Treg/CD4+ (%) ≥ 5.07 group (*P* = 0.0029). The vascular invasion rate was also significantly higher in the *EpCAM*^mRNA+^ CTC ≥ 2.22 group (*P* = 0.027), suggesting the occurrence of tumor microenvironmental changes in early recurrent cases in addition to pathological changes.

With the expansion of the tumor microenvironment, tumor cells spread from the primary lesions, thereby forming circulating tumor cells (CTCs). Thousands of CTCs are generated each day, but not all CTCs can become “seeds” of metastatic recurrence. Besides environmental factors, the inherent characteristics of CTCs are also crucial for metastasis [20]. In recent years, with the introduction of the “tumor stem cell” concept, tumor stem cells have been shown to exhibit stem cell-like features including high capacity for self-renewal, differentiation, and the generation of heterogeneous cells, as well as high resistance to chemotherapy, radiotherapy and cytotoxic agents, combined with high capacity for oncogenesis and tumor maintenance. There is sufficient evidence that a high ratio of tumor stem cell-like cells indicates a poor prognosis [21]. Sun et al. reported that *EpCAM*^mRNA+^ CTCs retaining stem cell-like characteristics are “high-quality seeds” for metastasis, and that the level of *EpCAM*^mRNA+^ CTCs is an ideal predictor for postoperative early recurrence and prognosis of HCC [22].

Tumor-related immune suppression is mediated mainly by increased TGF-β secretion or direct Treg cell infiltration [23]. A recent study [24] found an association between intratumoral or peripheral blood Tregs and tumor invasion. Tregs mediate tumor immune escape and promote tumor growth mainly by suppressing tumor immune effector cells (especially cytotoxic lymphocytes), or by inducing effector T cell tolerance to tumor antigens. The resulting imbalance between intratumoral Tregs and cytotoxic T cells was shown to be an effective prognostic predictor. In our study, a significant correlation was observed between the levels of *EpCAM*^mRNA+^ CTC and peripheral Treg/CD4^+^, with an increasing trend (*P* = 0.026). This result may supported that Tregs contributed as “soil” which may change the tumor microenvironment to help CTCs get out of immune clearance by cytotoxic T cells as well as colonization in HCC patients.

The results of univariate Cox analysis found that the significant prognostic factors for early recurrence included *EpCAM*^mRNA+^ CTC ≥ 2.22 (*P* = 0.001) and Treg/CD4 + ≥ 5.07 (*P* = 0.045). Further multivariate Cox analysis revealed *EpCAM*^mRNA+^ CTC ≥ 2.22 (*P* = 0.003, HR = 6.668) to be a significant and independent prognostic biomarker for early recurrence, in accordance with the study by Sun et al., which reported *EpCAM*^mRNA+^ CTC ≥ 2 to be an independent predictor for early HCC recurrence (within 1 year following resection) [13]. Survival curve analyses found that the early recurrence rates within the *EpCAM*^mRNA+^ CTC ≥ 2.22 group (12.5 % vs. 58.8 %, *P* = 0.002, Fig. 3a) and Treg/CD4^+^ ≥ 5.07 group (22.5 % vs. 55.6 %, *P* = 0.038, Fig. 3b) were markedly elevated. Combining these two factors of “soil” and “seeds”, we found that the early recurrence rate in the group with combined high CTC and high Treg levels was significantly higher than in the combined low CTC and low Treg group (66.7 % vs. 10.3 %, *P* < 0.001, Fig. 3c), while the recurrence rate within the combined high CTC and low Treg group was 46.4 % higher than for the combined low CTC and low Treg group (50.0 % vs. 10.3 %, *P* = 0.004, Fig. 3c). These results also implied that elevated Tregs cells could cause immune suppression, and contribute CTCs escape from peripheral immune clearance. Consequently, the spread of CTCs lead to HCC metastasis and recurrence. However, the mechanisms of *EpCAM*^mRNA+^ CTC and Treg cells interaction remain unclear, warranting future larger clinical studies as well as further basic explorative research.

The limitations of the current study were a small cohort size, short follow-up time, and only patients with Hepatitis-B induced HCC and early stages (BCLC 0 and A) have been included in this study, which may results the clinical significance of Edmondson classification and AFP in HCC were not be observed. These limitations will be addressed in our next step clinical investigation.

## Conclusions

In summary, our study found an association between peripheral blood *EpCAM*^mRNA+^ CTC and CD4^+^CD25^+^Foxp3^+^ Treg preoperative levels with postoperative recurrence and metastasis in HCC patients. Combined measurement of both cell types may improve prognostic efficacy. We are currently carrying out a large prospective randomized controlled trial to further confirm the value of this combined measurement in predicting HCC recurrence and prognosis.

## Abbreviations

CTCs, circulating tumor cells; HCC, hepatocellular carcinoma; Treg, T regulatory

## Additional files

Additional file 1: Table S1. Patient characteristics. (DOCX 16 kb) Additional file 2: Figure S1. Flow cytometry analysis for Tregs. The original data of flow cytometry analysis for Tregs. (PNG 766 kb) Additional file 3: Table S2. Association of lymphocyte subgroups with early recurrence. (DOCX 15 kb)
