# Supplementary material for: Association of preoperative EpCAM Circulating Tumor Cells and peripheral Treg cell levels with early recurrence of hepatocellular carcinoma following radical hepatic resection
Source: BMC Cancer. 2016 Jul 20;16:506. doi: 10.1186/s12885-016-2526-4 (PMC4955266; doi:10.1186/s12885-016-2526-4)
Supplement: Additional file 1: Table S1. — Patient characteristics. (DOCX 16 kb) [file 12885_2016_2526_MOESM1_ESM.docx]

**Supplement table 1. Patient characteristics**

| **Variable** | | **N** | **%** |
| --- | --- | --- | --- |
| Age (y) | ≤50 | 23 | 46.9 |
| Sex | Male | 13 | 26.5 |
| HBsAg | Negative | 9 | 18.4 |
|  | Postive | 40 | 82.6 |
| HBVDNA | <5*10^2^ | 31 | 63.2 |
|  | >5*10^2^ | 18 | 36.8 |
| HBeAg | Negative | 36 | 73.4 |
|  | Postive | 13 | 26.6 |
| Child-Pugh score | A | 48 | 98.0 |
|  | B | 1 | 2.0 |
| Liver cirrhosis | No | 12 | 24.5 |
|  | Yes | 37 | 75.5 |
| ALT (U/L) | ≤75 | 33 | 67.3 |
|  | >75 | 16 | 32.7 |
| AFP (ng/ml) | ≤400 | 39 | 79.6 |
|  | > 400 | 10 | 20.4 |
| Tumor number | Single | 44 | 89.8 |
|  | Multiple | 5 | 10.2 |
| Tumor size (cm) | ≤5 | 29 | 59.2 |
|  | >5 | 20 | 40.8 |
| Tumor encapsulation | Complete | 21 | 42.9 |
|  | None | 28 | 57.1 |
| Satellite lesion | No | 44 | 89.8 |
|  | Yes | 5 | 10.2 |
| Vascular invasion | No | 25 | 51.0 |
|  | Yes | 24 | 49.0 |
| Edmondson stage | I-II | 31 | 63.3 |
|  | II-IV | 18 | 36.7 |
| BCLC stage | 0+A | 44 | 89.8 |
|  | B+C | 5 | 10.2 |
