# Supplementary figures and images for: Association of preoperative EpCAM Circulating Tumor Cells and peripheral Treg cell levels with early recurrence of hepatocellular carcinoma following radical hepatic resection
Source: BMC Cancer. 2016 Jul 20;16:506. doi: 10.1186/s12885-016-2526-4 (PMC4955266; doi:10.1186/s12885-016-2526-4)

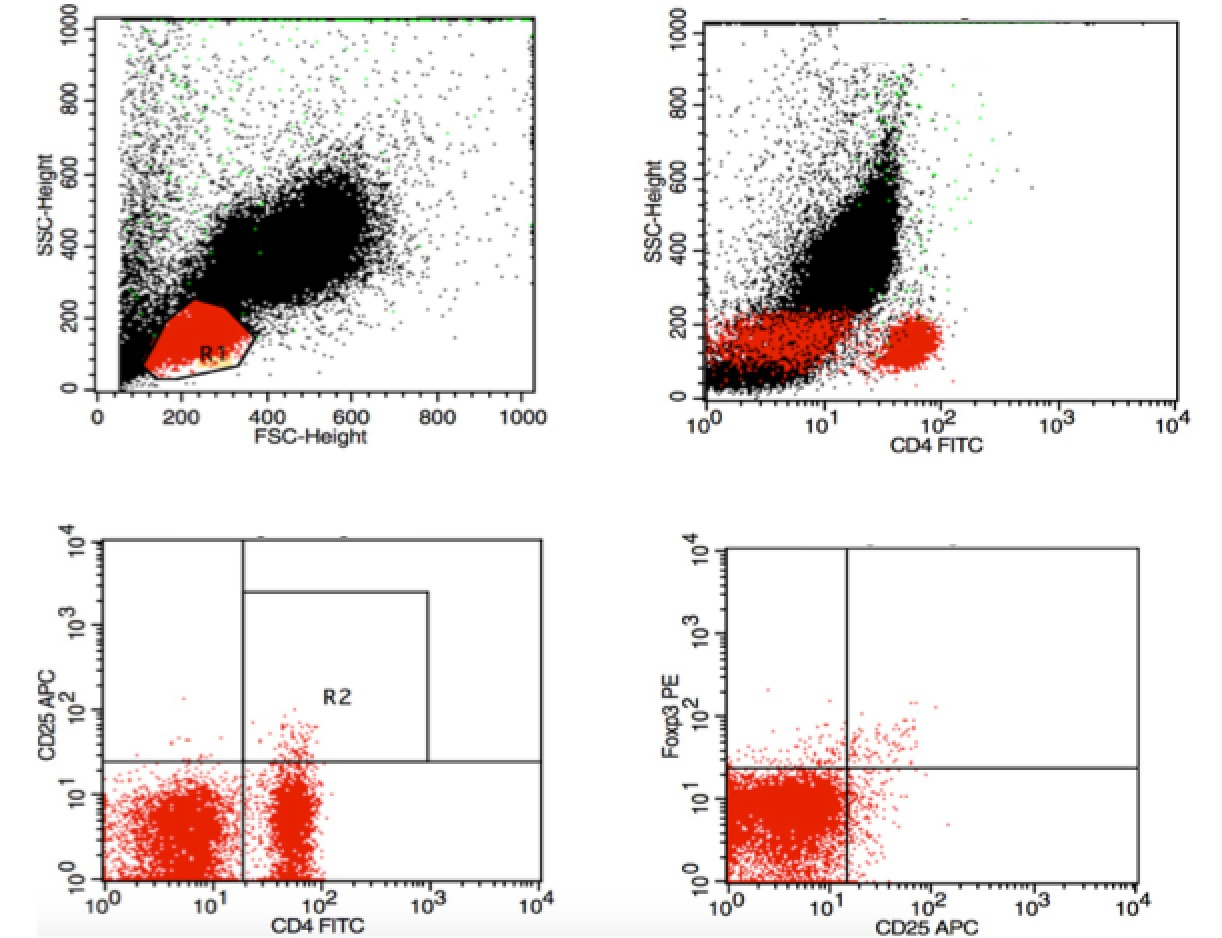

Supplement: Additional file 2: Figure S1. — Flow cytometry analysis for Tregs. The original data of flow cytometry analysis for Tregs. (PNG 766 kb) [file 12885_2016_2526_MOESM2_ESM.png]
