# Supplementary material for: Association of preoperative EpCAM Circulating Tumor Cells and peripheral Treg cell levels with early recurrence of hepatocellular carcinoma following radical hepatic resection
Source: BMC Cancer. 2016 Jul 20;16:506. doi: 10.1186/s12885-016-2526-4 (PMC4955266; doi:10.1186/s12885-016-2526-4)
Supplement: Additional file 3: Table S2. — Association of lymphocyte subgroups with early recurrence. (DOCX 15 kb) [file 12885_2016_2526_MOESM3_ESM.docx]

**Supplement table 2. Association of** **lymphocyte subgroups with early recurrence**

| Variables | | Early recurrence | | P |
| --- | --- | --- | --- | --- |
|  |  | No | Yes |  |
|  | CD19 (%) | 35 | 14 | 0.251 |
|  |  |  |  |  |
|  | CD3 (%) | 35 | 14 | 0.359 |
|  |  |  |  |  |
|  | CD4 (%) | 35 | 14 | 0.711 |
|  |  |  |  |  |
|  | CD8 (%) | 35 | 14 | 0.120 |
|  |  |  |  |  |
|  | CD16+56 (%) | 35 | 14 | 0.619 |
|  |  |  |  |  |
|  | CD4/CD8 | 35 | 14 | 0.353 |
|  |  |  |  |  |
|  | - Treg/lymphocyte (%) | 35 | 14 | 0.195 |
|  |  |  |  |  |
|  | Treg/CD8 (%) | 35 | 14 | 0.958 |
|  |  |  |  |  |
|  | Treg/CD4 (%) | 35 | 14 | 0.020* |
|  |  |  |  |  |
|  | Treg/CD3 (%) | 35 | 14 | 0.197 |
|  |  |  |  |  |
